# Analogues of Anticancer Natural Products: Chiral Aspects

**DOI:** 10.3390/ijms24065679

**Published:** 2023-03-16

**Authors:** Jindra Valentová, Lucia Lintnerová, Natalia Miklášová, Bianka Oboňová, Ladislav Habala

**Affiliations:** Department Chemical Theory of Drugs, Faculty of Pharmacy, Comenius University in Bratislava, Odbojárov 10, 832 32 Bratislava, Slovakia

**Keywords:** natural products, chirality, anticancer activity, semisynthetic drugs, enantiomers, chemotherapy

## Abstract

Life is chiral, as its constituents consist, to a large degree, of optically active molecules, be they macromolecules (proteins, nucleic acids) or small biomolecules. Hence, these molecules interact disparately with different enantiomers of chiral compounds, creating a preference for a particular enantiomer. This chiral discrimination is of special importance in medicinal chemistry, since many pharmacologically active compounds are used as racemates—equimolar mixtures of two enantiomers. Each of these enantiomers may express different behaviour in terms of pharmacodynamics, pharmacokinetics, and toxicity. The application of only one enantiomer may improve the bioactivity of a drug, as well as reduce the incidence and intensity of adverse effects. This is of special significance regarding the structure of natural products since the great majority of these compounds contain one or several chiral centres. In the present survey, we discuss the impact of chirality on anticancer chemotherapy and highlight the recent developments in this area. Particular attention has been given to synthetic derivatives of drugs of natural origin, as naturally occurring compounds constitute a major pool of new pharmacological leads. Studies have been selected which report the differential activity of the enantiomers or the activities of a single enantiomer and the racemate.

## 1. Introduction

Cancer is one of the leading causes of death globally, second only behind ischemic heart disease. Lung, prostate, colorectal, stomach, and liver cancer are the most common types of cancer in men, while breast, colorectal, lung, cervical, and thyroid cancer are the most common among women [1].

The primary treatment modalities encompass surgery, chemotherapy, radiation, immunotherapy, etc. However, the mainstay treatment is based on chemotherapy which employs various compounds of natural and synthetic origin that can kill cancer cells or stop their unwanted proliferation [2,3].

The compounds used in the chemotherapy of cancer disease are quite varied in structure and mechanism of action, comprising alkylating agents; antimetabolite analogues of folic acid, pyrimidine, and purine; natural products; hormones and hormone antagonists; and a variety of agents directed at specific molecular targets. The majority of anticancer agents interact with DNA or its precursors, inhibiting the synthesis of new genetic material and causing damage to DNA in both normal and malignant cells [4,5]. The rapidly expanding knowledge of cancer biology has brought about the discovery of entirely new and more cancer specific targets (e.g., growth factor receptors, intracellular signalling pathways, epigenetic processes, tumour vascularity, DNA repair effects, and cell death pathways [6,7].

Throughout history, natural products have been the basis of therapy for a variety of diseases. More than half of the currently used drugs are based on natural compounds [8]. Besides molecules isolated directly from natural sources and applied as such in therapy, this category encompasses also compounds derived from them using chemical methodology, as well as fully synthetic compounds which employ the natural compounds as structural models for the preparation of more efficient analogues. These derivatisation and modification strategies provide drugs with improved pharmacological activities and facilitate the overcoming of inherent drawbacks associated with many drug-like compounds isolated from natural sources, such as poor aqueous solubility and marked adverse reactions [9,10,11,12]. Thus, pharmacomodulation employs a natural compound with known biological activity as the origin, lead, prototype, or series head. Combinatorial chemistry in combination with high-throughput synthesis provides large libraries of bioactive compounds, making it possible to identify new lead molecules [13]. Manipulation of biosynthetic pathways constitutes another powerful tool for the preparation of derivatives of bioactive compounds [14]. Biosynthesis often affords structures not accessible through chemical synthesis due to their high structural and stereochemical complexity. Semisynthetic derivatives of natural products also play an important role in the development of prodrugs [15].

## 2. Anticancer Natural Products and Their Semisynthetic Congeners

Today, the fraction of anticancer drugs related in one way or another to natural sources amounts to over 60% [16,17]. Although in the 1990s they temporarily fell out of favour with commercial pharmaceutical research due to the emergence of targeted therapies, recently we have encountered a revived interest in this category of bioactive compounds. According to a study of new and approved drugs for cancer by the United States Food and Drug Administration (FDA), from 1940s–2010, of the 175 small molecules, 74.8% were other than synthetic [18].

**Figure 1:**
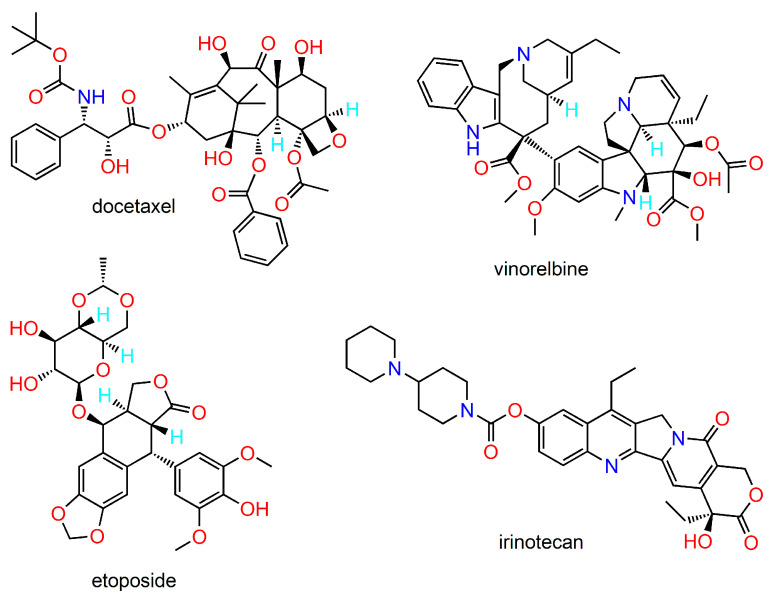
Examples of the main classes of antineoplastic drugs from plants.

Historically, (terrestrial) plants constitute the first major source of natural products. The main categories of anticancer natural compounds of herbal origin (including their semisynthetic derivatives) comprise taxanes, vinca alkaloids, camptothecins, and podophyllotoxins. Representative members of these drug classes are shown in Figure 1.

Taxanes are among the most important chemotherapeutic agents in clinical use [19,20,21]. They belong to the group of microtubulin-stabilising agents. The parent compound of the taxane class is paclitaxel, isolated from the bark of the Pacific yew tree (*Taxus brevifolia*). Docetaxel is a semisynthetic analogue with improved anticancer activity along with better pharmacokinetic properties. A number of structural analogues have been developed with a view to overcome the limitations of paclitaxel and docetaxel [22].

Podophyllotoxin is a lignin isolated from the roots and rhizomes of *Podophyllum* species. It has a long history of use in traditional medicine for various indications. Podophyllotoxin inhibits the polymerisation of tubulin, destabilising microtubules and preventing cell division. Its use in antineoplastic therapy is impaired especially by low bioavailability and high toxicity. Thus, several semisynthetic analogues have been developed, the most successful of them being etoposide, teniposide, and etopophos [23,24]. They are irreversible inhibitors of topoisomerase II, inducing DNA cleavage.

Vinca alkaloids are microtubulin-disrupting agents, originally isolated from the periwinkle plant *Catharanthus roseus*. Most important among them are vinblastine and vincristine, efficacious anticancer drugs in clinical practice [25]. Numerous semisynthetic analogues have been developed, including vinorelbine, vindesine, vincamine, and vinflunine [26]. These particular compounds show marked differences in their spectrum of activity as well as toxicity profiles [27]. Consequently, they have differing clinical application areas in antineoplastic therapy.

Camptothecin is an alkaloid found in the bark of the Chinese tree *Camptotheca acuminata*. Its anticancer activity relates to the inhibition of topoisomerase I via the formation of a ternary complex between the enzyme, DNA, and camptothecin, preventing DNA relegation [28]. Despite its marked antineoplastic effect, its clinical utility is limited due to severe adverse reactions, as well as unsatisfactory solubility and bioavailability [29]. In order to improve its pharmacological profile and to reduce the side effects, many semisynthetic analogues have been prepared and evaluated [30,31]. Thus, irinotecan and topotecan have found their way into clinical practice; other examples include belotecan, silatecan, cositecan, exatecan, lurtotecan, and rubitecan.

**Figure 2:**
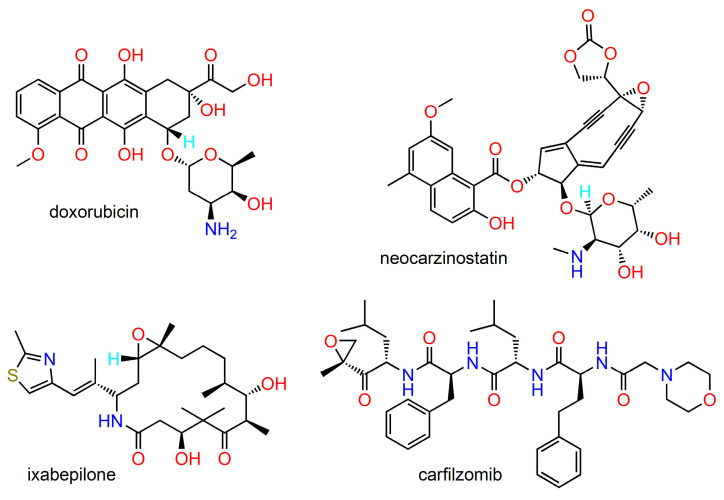
Representatives of antitumour antibiotics.

Microbial-based compounds are among the oldest and most important chemotherapeutic agents in use, including bleomycin, actinomycin, ansamycin, anthracyclines, epothilones, and enediynes, among others [32]. Examples of their structures are given in Figure 2. A substantial majority of antitumour antibiotics originate from various *Streptomyces* species. A wide variety of structural analogues with improved pharmacological profiles can be obtained by a combination of genetic engineering techniques and methods of organic synthesis [33]. A modern and useful technique for the identification of new microbial secondary metabolites is genome mining [34]. Recently there has been increasing interest in the discovery of new cytotoxic compounds from unconventional sources, such as plant-associated microorganisms or marine habitats.

**Figure 3:**
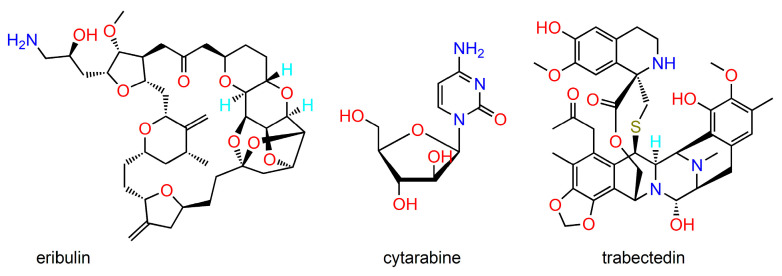
Selected structures of marine compounds with antineoplastic activity.

Despite the enormous biodiversity of marine organisms, only a small fraction of marine habitats has been pharmacologically explored. Recent advancements in isolation and purification techniques, structure elucidation, synthetic modification, and biological assays rendered possible the isolation and pharmacological evaluation of numerous unique anticancer compounds from ocean habitats [35,36,37]. In this regard, a major source of anticancer compounds are various marine sponges. In addition, diverse organisms such as molluscs, tunicates, algae, marine microbes, and various chordates can be sources of bioactive agents of astounding structural diversity [38,39]. Figure 3 shows the structures of selected marine-derived molecules with antineoplastic activity. Cytarabine is a synthetic drug modelled after the natural compound found in the Caribbean sponge *Tectitethya crypta*. Trabectedin is an antitumour chemotherapeutic drug discovered in the extract from the sea squirt *Ecteinascidia turbinata*. Eribulin is a synthetic analogue of the marine natural product halichondrin B (found in the sponge *Halichondria okadai*), both compounds being potent mitotic inhibitors. An interesting anticancer agent is brentuximab vedotin, a semisynthetic bioconjugate prepared from the chimeric monoclonal antibody brentuximab and monomethyl auristatin E, a synthetic analogue derived from dolastatins, natural peptides occurring in the marine mollusc *Dolabella auricularia*.

## 3. Chirality in Drug Design

Stereoisomers are compounds which differ only in the three-dimensional arrangement of their constituent atoms in space. Such isomers may be divided into two groups—enantiomers and diastereoisomers. Enantiomers are pairs of compounds which are non-superimposable mirror images of each other and, in terms of physicochemical properties, differ only in their ability to rotate plane polarised light. Such isomers are called chiral and are referred to as optical isomers. Diastereoisomers are stereoisomers which do not appear as mirror images of each other. They can be chiral or geometrical (*cis*/*trans*) isomers. A mixture of equal quantities of two enantiomers is called racemate or racemic mixture.

**Figure 4:**
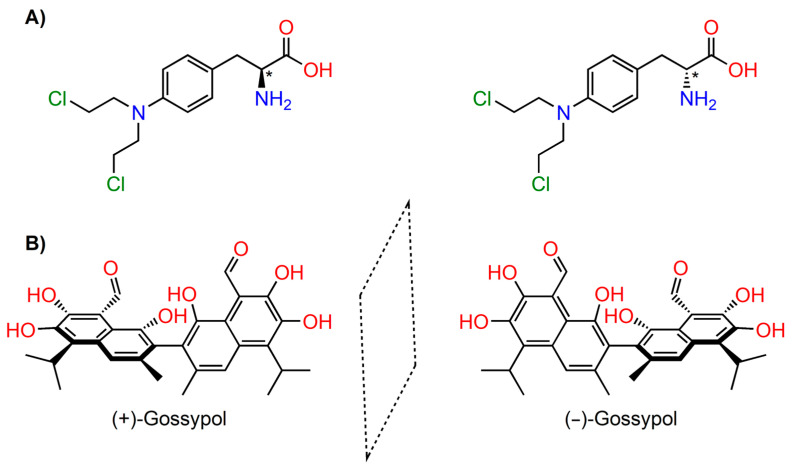
Examples of enantiomeric pairs in the group of chiral anticancer compounds [43,44]. (**A**) Melphalan carrying the stereogenic centre (marked with asterix)—carbon with four different atoms or groups. (**B**) Gossypol stereoisomers resulting from atropoisomerism. Stereoisomers are denoted according to the configuration of stereogenic centre (*R*)- (*S*)-, or their optical activity (+), (−).

Chirality is a property inherent to all biological systems. Biomacromolecules composed of simpler chiral subunits (amino acids, sugars, lipids) fold into complex three-dimensional architectures, exhibiting supramolecular chirality. Chiral macromolecular scaffolds contain asymmetric binding sites and catalytic centres capable of recognising and discriminating between individual stereoisomers of other chiral molecules [40,41,42]. Most often, chirality arises from the presence of asymmetric centres in organic molecules; generally, these are tetracoordinate centres to which four different atoms or group are connected, such as in the chiral chemotherapy agent melphalan (Figure 4A). Much less frequently, chirality is caused by atropoisomerism, i.e., the hindered rotation about a single bond, e.g., ortho-substituted biphenyl derivatives are chiral due to restricted rotation; an interesting example is gossypol, a bioactive yellow pigment of natural origin (Figure 4B).

**Figure 5:**
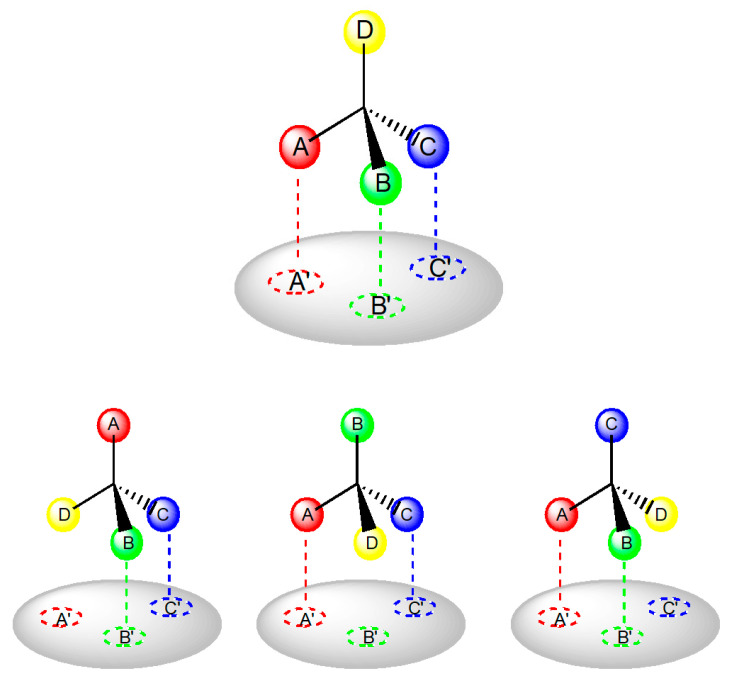
Interaction of a chiral drug with the receptor site [45]. The more active enantiomer is capable of achieving the correct orientation to enable all three functional groups to fit their respective binding sites on the receptor active sites A...A′, B....B′, C...C′. The less active enantiomers may interact at two sites only, regardless of its orientation towards the active site.

Many biochemical processes during drug action require interaction with chiral biomolecules, hence it is not surprising that enzyme and receptor systems frequently exhibit a stereochemical preference towards one of a pair of enantiomers. Enantiomers may differ both quantitatively and qualitatively in their biological activities. The Easson Stedman hypothesis is generally used to explain the difference in the biological activity of enantiomers. It asserts that the difference in activity is caused by differential binding of the pair of enantiomers to the common binding site [45]. The more active enantiomers must be involved in a minimum of three intermolecular interactions with the receptor surface; the less potent enantiomer only interacts with two sites (Figure 5). In some cases, especially in the interaction of drugs with enzymes, it is asserted that a fourth location, either a direction requirement or an additional binding site is essential to discriminate between the enantiomers [46].

The differential pharmacodynamic and toxicological properties of the enantiomers of chiral drugs have been known for a number of years, affecting essentially all categories of drugs [47,48,49,50,51]. At one extreme, one enantiomer may be devoid of any biological activity; at the other extreme, both enantiomers may exhibit qualitatively different biological activities. Furthermore, the required activity may reside in both enantiomers, but the adverse effects can be predominantly associated with only one enantiomer, or the enantiomers may have opposite effects on the same biological target [52]. These stereoselective differences may arise not only from drug interactions at the pharmacological receptors but also from pharmacokinetic events [53]. Differences between enantiomers may occur during their absorption, distribution, metabolism, and excretion. Thus, following the administration of a racemic drug, the individual enantiomers do not reach their site of action in equal concentrations [54,55].

As a result of advances in chemical techniques, especially in the methodology of stereoselective syntheses and stereospecific analyses, together with regulatory requirements, the number of chiral drugs submitted for approval to regulatory authorities as single enantiomers rather than racemates has increased considerably [56]. Compared to the end of the last century, when about 55% of clinically used drugs were chiral and half of them were used as racemates, the current trend in the development of new drugs is mainly towards substances containing one single enantiomeric form [57,58]. In addition to new chemical entities, a number of established racemic drugs have been re-evaluated as potential single enantiomer products with the possibility of an improved therapeutic profile or application in other therapeutic indications. As an example of this “chiral switching“ concept, the originally racemic nonsteroidal anti-inflammatory drugs (e.g., ibuprofen, ketoprofen) were marketed as single (*S*)-enantiomers, since this enantiomer is mainly responsible for the antiphlogistic effect [59]. Several studies demonstrated the association of these anti-inflammatory drugs with decreased cancer incidence and recurrence [60,61]. Interestingly, the (*R*)-enantiomer of flurbiprofen has been found effective against colon and prostate cancer as well as against formation of glioblastomas in in vitro and in vivo models [62,63,64]. Investigating the influence of varying configuration at the chiral centre on biological effects is rapidly becoming a significant part in the discovery of novel chemotherapeutic agents [55,65].

Chirality is one of the important factors that determine the structure of a particular anticancer drug and its interaction with cancer molecular targets. This is true not only for purely organic drugs but also for metal-based drugs developed for anticancer applications [66]. The chiral metal-based anticancer drugs have been comprehensively reviewed in the literature [66,67] and the influence of chirality on the antineoplastic effect of synthetic organic compounds was surveyed by Valentova et al. [68]. The antitumour activity of natural and synthetic chiral flavonoids is the subject of a literature review by Pinto et al. [69].

This review deals with recent studies on diverse chiral antineoplastic agents exhibiting different mechanisms in their anticancer effects. We focus on selected examples of chiral natural anticancer compounds and their analogues to provide the reader with an overview of the most important developments. Only studies which report the anticancer activities of both enantiomers, or a comparison between a single enantiomer and the racemate are considered, thus allowing the assessment of the influence of stereochemical arrangement on antitumour activity.

## 4. Chiral Analogues of Natural Compounds with Stereospecific Cytotoxic Activity

### 4.1. Chiral Xanthones

**Figure 6:**
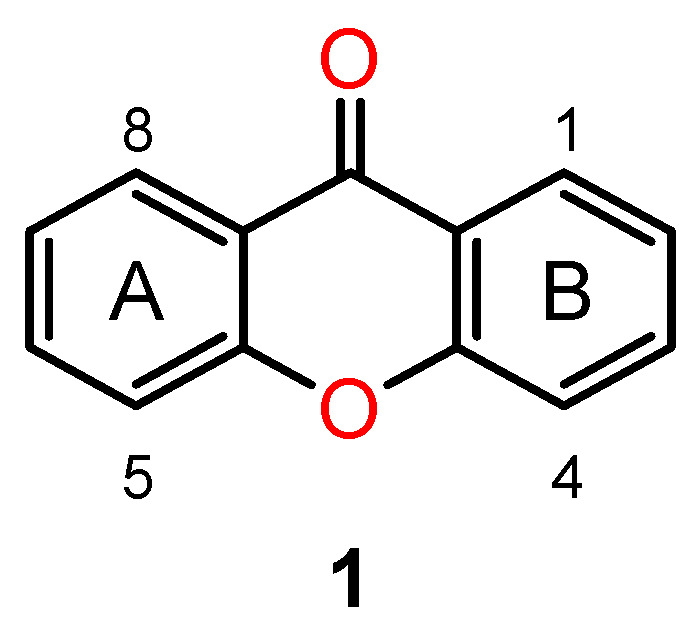
The basic scaffold of the xanthone class of compounds.

Many naturally occurring xanthones, those isolated from plants as well as marine sources, are chiral and exhibit interesting biological activities [70,71,72]. Chemically, xanthones are compounds with an oxygen-containing dibenzo-pyrone heterocyclic scaffold—9H-xanthen-9-ones (**1**) (Figure 6). Within this class of compounds and their synthetic derivatives, the main biological activities reported have been antitumour and antimicrobial activities [73,74].

The renowned *Garcinia* plants widespread in tropical zones of Asia, Australia, and America are a major source of natural polyprenylated xanthones and benzophenones with significant antitumour activity [75]. Different mechanisms can play a role in the cytotoxic effects of xanthones, including the induction of apoptosis, cell proliferation arrest and autophagy, and inhibition of telomerase. They also demonstrated antimetastasis, anti-angiogenesis and anti-inflammatory activities [71].

**Figure 7:**
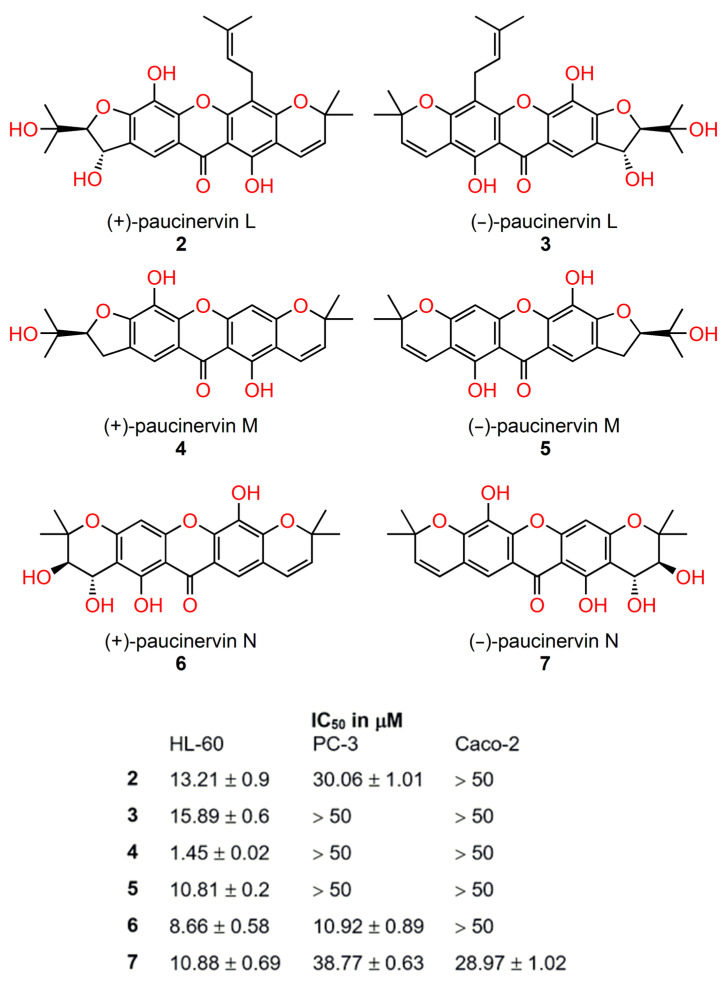
The structure and cytotoxic activity of the new chiral paucinervin enantiomers with an antiproliferative effect [77].

Several *Garcinia* species are important in local medicine, and some are cultivated for their fruit or as ornamentals [76]. Recently, three pairs of newly discovered polyisoprenylated xanthone enantiomers, (±) paucinervins L, M, N (**2**–**7**) (Figure 7) and two new xanthones, (−) paucinervin O and paucinervin P, along with thirteen known xanthones were isolated from the stem of *Garcinia paucinervi* [77]. All isolated xanthones were evaluated for anticancer activity against the myeloid–promyelocytic cell line HL-60, the human prostate cancer cell line PC-3, and the colon adenocarcinoma cell line Caco-2 [77]. Enantiomeric pairs of paucinervins L-N exhibited the strongest antiproliferative effects against the HL-60 cell line (IC_50_ in the range 0.8–8 μM). Interestingly, the xanthones with dextro-rotation (+) showed more a potent effect that those with laevo-rotation (−). In the case of paucinervin M, the (−)-enantiomer was ten times more cytotoxic than the (+)-enantiomer.

**Figure 8:**
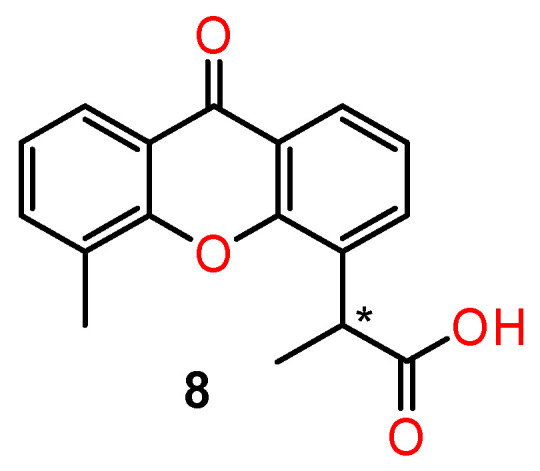
The structure of chiral analogues of dimethyl xanthone-4-acetic acid. * stereogenic centre.

Differences in antitumour activity of the chiral synthetic derivatives of xanthones were reviewed by Fernandez et al. [78]. The synthetic analogues of xanthone-4-acetic acid, one of the most studied xanthones with regard to its pharmacological activities, are worth mentioning [79,80]. The dimethyl analogue of xanthone-4-acetic acid is a tumour vascular-disrupting agent leading to vascular collapse and tumour necrosis by immunomodulation and the action of cytokines. [81]. Its chiral analogues (**8**) (Figure 8) exhibited enantioselectivity in their antitumour activity—the ability to cause early haemorrhagic necrosis of colon tumours in mice. The (*S*)-(+) enantiomer of 5-methyl-α-xanthone-4-acetic was much more dose-potent than the (*R*)-(−) enantiomer in both in vitro and in vivo tumour assays. This suggests that the enantiomers have different intrinsic activities rather than differing in their in vivo metabolism [79].

### 4.2. Chiral Baicalin

**Figure 9:**
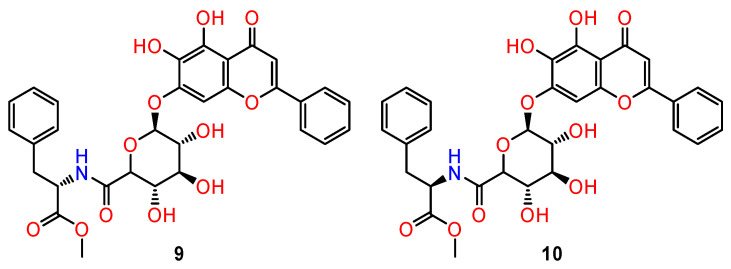
The structures of chiral baicalin derivatives formed from baicalin and phenylalanine methyl esters-BAL (**9**) and BAD (**10**).

The different anti-neoplastic activities in different cell lines were also demonstrated with the chiral derivatives of baicalin, a flavonoid extracted from *Scullaria baicalensis* Georg, and used as a potential antitumour active ingredient in Chinese traditional medicine [82,83]. Chiral derivatives of baicalin were prepared by combining baicalin with either D- or L-phenylalanine methyl ester [84]. Antitumour activities of chiral derivatives of baicalin—BAL (**9**) (derived from L-phenylalanine methyl ester) and BAD (**10**) (derived from D-phenylalanine methyl ester) (Figure 9) were investigated against lung (A549, H460, Calu-1) and breast cancer cell lines (MBA-M-435, MCF-7, T47D) in in vitro and in vivo studies. The prepared derivatives had a stronger inhibitory effect on lung cancer cell lines, especially on the A549 cell line, compared with pure baicalin. The antiproliferative activity of BAL was more remarkable than that of BAD. The inhibition rates of 50 mg/mL BA, BAD, and BAL on A549 cells at 48 h were 31.1%, 88.9%, and 94.1%, respectively.

In breast cancer lines, both BAL and BAD exhibited stronger inhibitory activity in T47D cells compared with baicalin. In contrast, BAL and BAD did not inhibit the proliferation of MDA-M-435 cells and exhibited inhibition in MCF-7 cells only at high concentrations.

BAL and BAD had a good inhibitory effect on subcutaneous tumour growth in nude mice in in vivo experiments, and the effect was shown to follow the order BAL > BAD > baicalin, which was consistent with the results in vitro. The higher antitumour activity of BAL compared with BAD and baicalin was related to the promotion of apoptosis of tumour cells via the phosphatidylinositol 3-kinase signalling pathway [84].

### 4.3. Chiral Derivatives of Ricinoleic Acid

(*R*)-(Z)-ricinoleic acid (RA) (**11**–**12**) is a natural fatty acid and is the main component of castor oil from *Ricinus communis* L., seeds. Many synthetic derivatives of RA with interesting biological activities have been obtained [85]. In particular, amides, esters, and glycosides exhibited potent antiproliferative and cytotoxic activities [86,87].

The modification of the parent compounds by amines resulted in increased cytotoxicity of the obtained products against HT29, HCT116, MCF-7, and AGS cancer cells (human colorectal adenocarcinoma cell line, human colorectal carcinoma cell line, human breast adenocarcinoma cell line, and human gastric adenocarcinoma cell line, respectively). The antitumour effect was observed for both enantiomeric forms. The most promising cytotoxic effects in terms of anticancer potential were obtained for ethanolamine-derived amides (**13**–**14**) [88].

**Figure 10:**
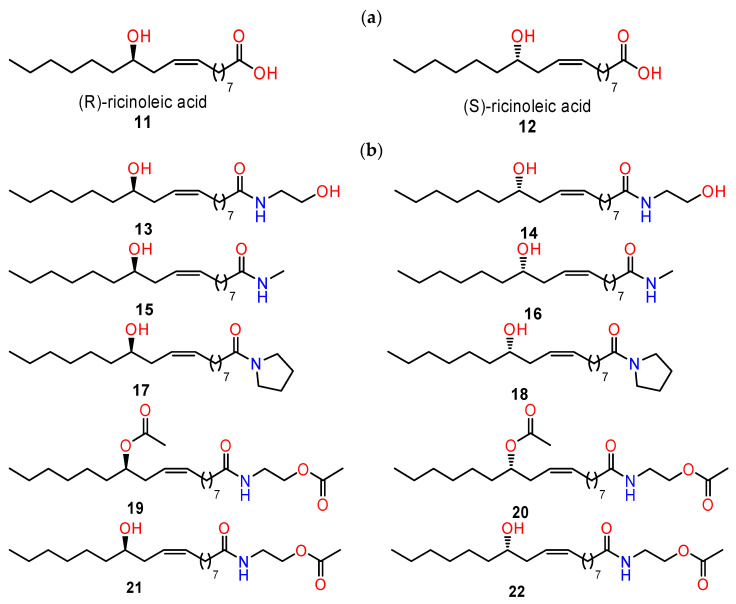
Chemical structures of enantiomeric forms of ricinoleic acid (**a**) and selected chemical derivatives (**b**).

Blaszczyk and co-workers [89] reported the synthesis and cytotoxic activity of both (*R*)- and (*S*)-enantiomers of ricinoleic acid amides and their acetates. The ricinoleic acid amides as well as acetate derivatives of ethanolamine amides were studied (**15**–**22**) (Figure 10) to demonstrate the influence of the stereogenic centre on their potential anticancer activity. The cytotoxic effect of the prepared compounds was evaluated against several cancer cell lines (HT29, HTC116, AGS, MFC7). Subsequently, the mechanism of cytotoxicity by the prepared enantiomers of RA-amide derivatives was evaluated using HT29 cancer cells. The ability to induce oxidative stress, DNA damage, and apoptosis was tested. Prepared compounds caused DNA damage and induced apoptotic and necrotic cell death. In most cases, only slight differences between the activities of the two enantiomers were observed. In the case of (*R*)- and (*S*)-enantiomers of one of the tested acetates (**21**,**22**), a significant difference in the ability to induce DNA damage was observed, which showed the impact of the stereogenic centre on the activities of these compounds [89].

### 4.4. Chiral Anthramycin Derivatives

Derivatives of anthramycin belong to antibiotics produced by various actinomycetes. Their selective cytotoxic activity towards tumour cells makes them a possible source of anticancer agents [90,91].

**Figure 11:**
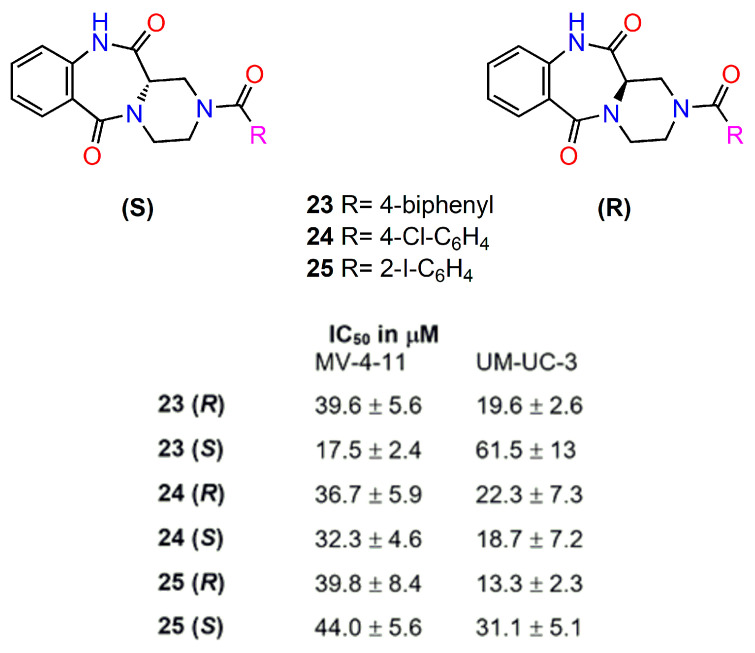
The structure and cytotoxic activity of the most active enantiomers of anthramycin analogues [92].

Mieczkowski et al. [92] reported the synthesis of novel chiral anthramycin analogues possessing a fused piperazine ring instead of a pyrrole and evaluated their cytotoxic activity in several cancer cell lines (Figure 11). Some of them were prepared as enantiomerically pure (*S*)- and (*R*)- isomers and were tested as single enantiomers for their antiproliferative potential on human biphenotypic B myelomonocytic leukaemia (MV-4-11) and human urinary bladder (TCC-UM-IC-3) cell lines. Cisplatin was used as a positive control (IC_50_ was 0,4 and 4,8, respectively). Most of the tested compounds showed similar cytotoxic effects in both cell lines (IC_50_ in the range of 10–44 μM). A significant difference between enantiomers was observed only in the case of (*S*) and (*R*) isomers of the derivative with a biphenyl substituent (**23**–**25**). The (*S*)-configuration at the chiral centre and the presence of a hydrophobic 4-biphenyl substituent were determined as key structural features responsible for the cytotoxic effect. Cell cycle arrest at the G1/S checkpoint and apoptosis associated with production of reactive oxygen species were also encountered in the most effective compounds [92].

### 4.5. Derivatives of Tetrahydroquinolin-8-Amines

Substituted tetrahydroquinolins are important structures present in a wide variety of natural alkaloids and synthetic analogues with high biological activity as potent antitumour agents [93,94]. Amino-quinoline derivatives have been reported to have antiproliferative activity due to their ability to induce mitochondrial dysfunction by increasing ROS levels in the sensitive cervical epithelioid carcinoma cell line HeLaS3 and in the multi-drug resistant human cervical cancer KB-vin cell line [95,96].

Based on these findings, Facchety et al. [97] investigated a new series of chiral derivatives of 2-methyl-5,6,7,8-tetrahydroquinolin-8-amine for their cytotoxic activity against a panel of human cancer cell lines: human T-lymphocyte (CEM), cervix carcinoma (HeLa), and dermal microvascular endothelial (HMEC-1) cells, as well as colorectal adenocarcinoma (HT-29), ovarian carcinoma (A2780), and biphasic mesothelioma (MSTO-211H) cells.

**Figure 12:**
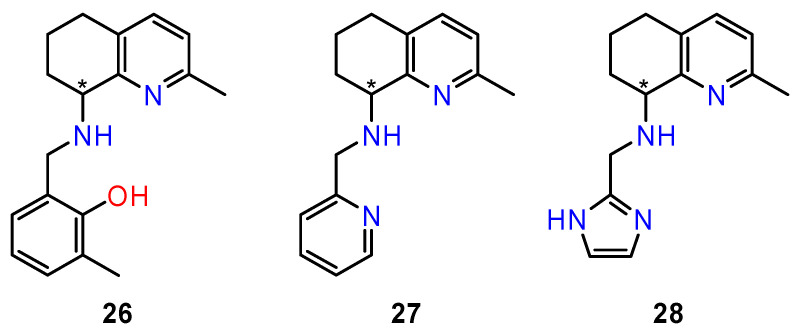
Structures of the tested tetrahydroquinoline amine derivatives. * stereogenic centre.

The influence of spatial arrangement of compounds on their biological effect is sometimes difficult to predict. In the case of chiral tetrahydroquinolin derivatives, the cytotoxic effect of enantiomers manifested differently depending on the type of cancer cells. In order to evaluate the different interaction of each enantiomer with their biological targets, the active compounds in the series were synthesised in an enantiomerically pure form—metylphenol derivative (**26**); pyridine derivative (**27**), and imidazole derivative (**28**) (Figure 12). Both enantiomers of prepared compounds were evaluated for their in vitro antiproliferative activity in three human tumour cell lines (HT-29, A2780, and MSTO-211H). All enantiomers showed a marked antiproliferative activity in A2780 cells (IC_50_ 5.4–17.2 μM). Remarkable differences between biological activities of the enantiomers were found in imidazole derivatives. The most effective was (*R*)-**28** and the least active was (*S*)-**28**.

Conversely, the two chiral forms of metylphenol and pyridine derivatives did not show any difference in terms of IC_50_, suggesting a similar cytotoxic effect. This behaviour was also confirmed in MSTO-211H cells and indeed, a comparable cytotoxic effect was observed in cells incubated with (*R*)-**27** and (S)-**27**, while both (*S*)-**26** and (*R*)-**26** were inactive in this cell line. On the other hand, the (*R*)-**28** enantiomer, unlike (*S*)-**28**, which appears ineffective, induced an appreciable inhibition of cell growth. Regarding colorectal adenocarcinoma cells (HT-29), they appeared resistant towards all synthesized compounds (IC_50_ > 20 μM). For the most active pyridine derivative, (*R*)-**27**, the mechanism of the cytotoxic effect was investigated. The compound was able to affect cell cycle phases and to induce mitochondrial membrane depolarisation and cellular ROS production in A2780 cells [97].

## 5. Inhibitors of Microtubule Polymerisation

### 5.1. Combretastatin A-4 Analogues

**Figure 13:**
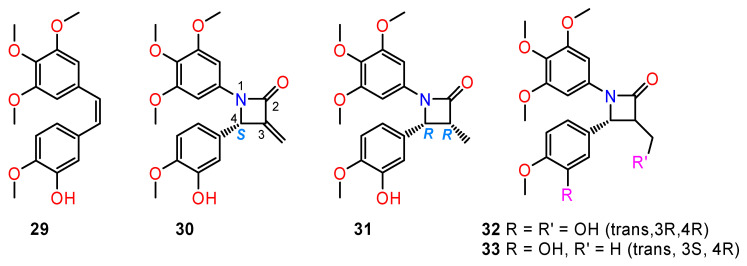
The structures of combrestatin (**30**) and its selected derivatives with strong tubulin-inhibitory activity (**31**–**34**).

Microtubules are important components of the cytoskeleton formed by polymerisation of the α- and β-subunit. Microtubules play a role in separating the daughter chromosomes to opposite poles during mitosis. The disruption of microtubules will result in the interruption of mitosis and leads to apoptosis of the cells [98]. Natural compounds such as colchicine (isolated from *Colchicum autumnale*) and combretastatin CA-4 (**29**) (isolated from the bark of the African tree *Combretum caffum*) with strong tubulin inhibitory activity served as templates for preparing more potent synthetic derivatives (**30**–**33**) (Figure 13) [99,100].

Zhou and co-workers [99] presented the synthesis and biological evaluation of diverse chiral β-lactam-bridged combretastatin A-4 analogues. In the cytotoxicity studies, the majority of the prepared target compounds displayed moderate to potent anti-proliferative activities against four human cancer cell lines (A2780, Hela, SKOV-3, and MDA-MB-231). The studies of structure–activity relationships revealed that the absolute configurations of the chiral C-4 atoms were critically important for the activity; more specifically, the (*S*)-configuration for 3-methylene-substituted series and the same orientation for other analogues. On this basis, *trans*-configuration of substituents at the 3,4-positions of the β-lactam scaffold benefits the antiproliferative activity. Among all the synthesised compounds, derivatives (**32**) and (**33**) turned out to be the most potent and were selected for further pharmacological studies. The co-crystal structures of tubulin in complex as determined by X-ray crystallography showed that derivatives (**32**) and (**33**) bind to the same site as colchicine with a similar binding mode.

### 5.2. Analogues of 4-Arylisochromenes

**Figure 14:**
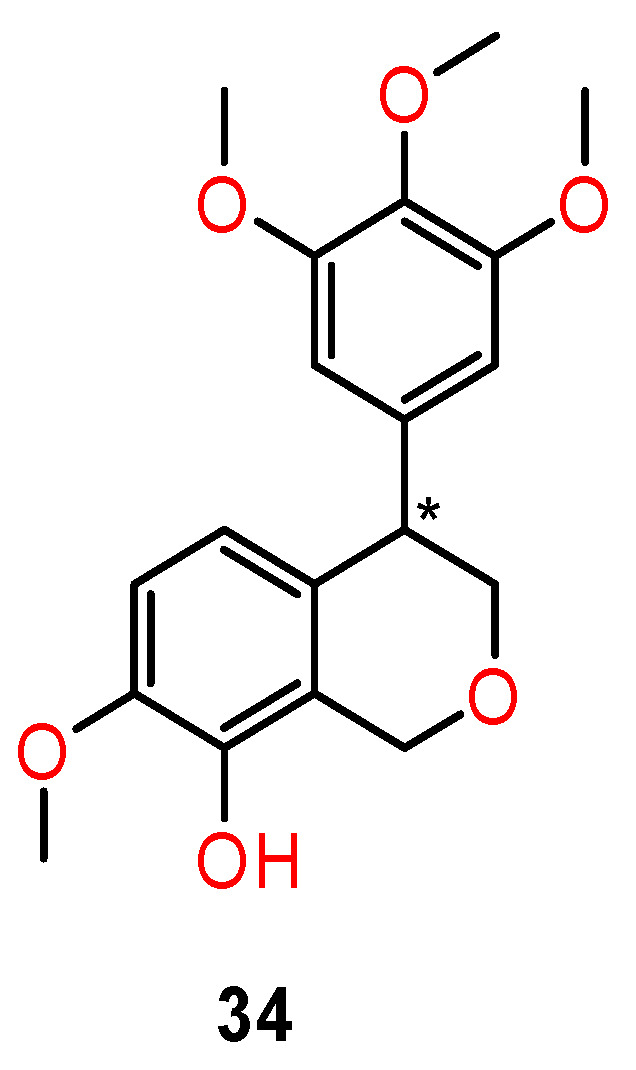
Structure of a chiral derivative of natural 4-arylisochromenes as an inhibitor of tubulin polymerisation. * stereogenic centre.

Weak inhibitory activity against tubulin polymerisation was also found in the 4-arylisochromenes derivatives isolated from the peel of *Musa sapine* tum. L (banana) [101]. Li et al. [102] prepared more effective chiral 4-arylisochromenes (**34**) (Figure 14) which are structural analogues of the natural inhibitor (±)-7,8-dihydroxy-3-methylisochroman-4-one.

Antiproliferative activity of prepared compounds was manifested against a panel of cancer cells: epithelial carcinoma (KB), ileocecal adenocarcinoma (HCT-8), breast cancer (MDA-MB-231), chronic myelogenous leukaemia (K562), and hepatocellular carcinoma (H22) cells, with IC_50_ values ranging from 10 to 25 nM. The racemic form and the (*R*)-enantiomer (**34**) were the most active against the K562 cell line with an IC_50_ value of 10 nM, which was more potent than the combretastatin (CA-4) (IC_50_ = 15 nM) used as a positive control, whereas the (*S*)-enantiomer (**35**) displayed a significant decrease of activity (IC_50_ = 460 nM).

**Figure 15:**
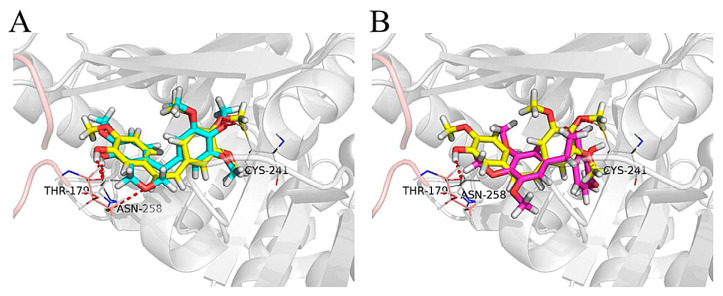
Proposed binding models for two *4-arylisochromenes* enantiomers (**35**) with tubulin (PDB code: 5lyj). (**A**) Combretastatin (shown in yellow); (*R*)-enantiomer (shown in blue). (**B**) CA-4 (shown in yellow); (*S*)-(−)-enantiomer (shown in violet). Reprinted with permission from Ref [102]. Copyright ©2018, American Chemical Society.

Chiral isomers of 4-arylisochromenes (**34**) also showed potent inhibitory activity against tubulin polymerisation. The (*R*)-enantiomer was slightly more potent than racemate, whereas the (*S*)-enantiomer displayed a significantly lower activity. The difference in activity for single enantiomers was illustrated by molecular modelling studies with tubulin crystal structures (PDB, 5lyj). The (*R*)-(+)-isomer exhibited very similar positioning with that of CA-4. The phenolic hydroxyl and 4-methyl groups of the (R)-enantiomer and CA-4 formed hydrogen bonds with Thr179 and Cys241 residues, respectively. The oxygen atom in the isochromene ring interacted with the Asn258 residue by a weak hydrogen bond. On the other side, the binding pose of the (*S*)-enantiomer was flipped over 180° compared with that of CA-4, which may explain why both the antitubulin and antiproliferative activity of the (*S*)-enantiomer decreased dramatically (Figure 15) [102].

### 5.3. Taxol Isomers

Nowadays, molecular docking is important in the investigation of the interaction between ligands and proteins and is among the most basic strategies for drug discovery. Molecular docking studies of interactions between active ligands and β-tubulin proteins have been utilised in the search for the most active chiral paclitaxel isomers. Paclitaxel (trade name Taxol^®^) is active in breast, ovarian, lung, bladder, prostate, melanoma, oesophageal, and other types of solid tumour cancers [103,104,105]. The molecule exerts its anticancer activity by inhibiting mitosis through enhancement of the polymerisation of tubulin and consequent stabilisation of microtubules [106,107].

**Figure 16:**
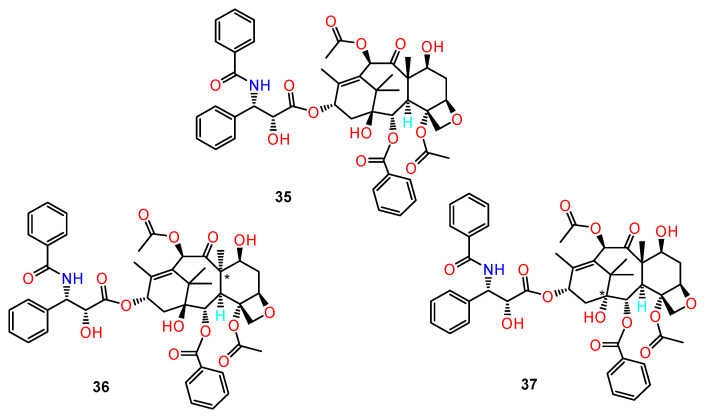
The structure of Taxol (**35**) and its diastereoisomers (**36**–**37**) with high affinity to β-tubulin according to molecular modelling studies. * stereogenic centre.

Paclitaxel is produced by extraction from the bark of yew trees (*Taxus brevifolia*), which grow very slowly. Therefore, obtaining paclitaxel from natural sources is not sufficient, and this has prompted extensive searches for alternative sources, including semisynthesis, cellular culture production and chemical synthesis. Taxol contains 11 chiral centres which makes it a very difficult target for total synthesis (Figure 16).

Ghadari et al. [108] investigated the effect of variations of chiral centres of Taxol (**35**) on the binding to β-tubulin by molecular modelling methods. They studied the hypothetical Taxol ligands which have been obtained by changing the configuration of atoms on one of the chiral centres. The binding activities of 12 different diastereoisomers were compared to the activity of the original Taxol structure.

In docking studies, the structures with better binding towards the protein were selected for further investigation using molecular dynamic simulation methods. The results showed that the structures with reversed configuration on the 5 and 8 chiral centres (**36,37**) have better affinity towards β-tubulin in comparison with Taxol and are thus good candidate compounds for further experimental studies. Derivatives with reversed configurations 1, 3, and 9 have similar affinity towards β-tubulin in comparison with Taxol. This work provides new opportunities for simplifying future preparation of synthetic analogues of Taxol by omitting the chiral centres which are not essential for the anticancer activity.

### 5.4. Maytansinoids

Significant differences in antitumour activity of enantiomeric forms based on interaction with microtubules were seen in chiral maytansinoids, the synthetic derivatives of maytansine. This compound was originally isolated from the African shrub *Maytenus ovatus* and belongs to the most potent microtubule inhibitors [109]. Some maytansinoid structures have been prepared in order to be linked to monoclonal tumour-specific antibodies [110]. It has been reported that maytansinoids with an L-configuration of the methyl group at the C3 position exhibited 100–400-fold higher antitumour activity than those with a D-configuration [111].

**Figure 17:**
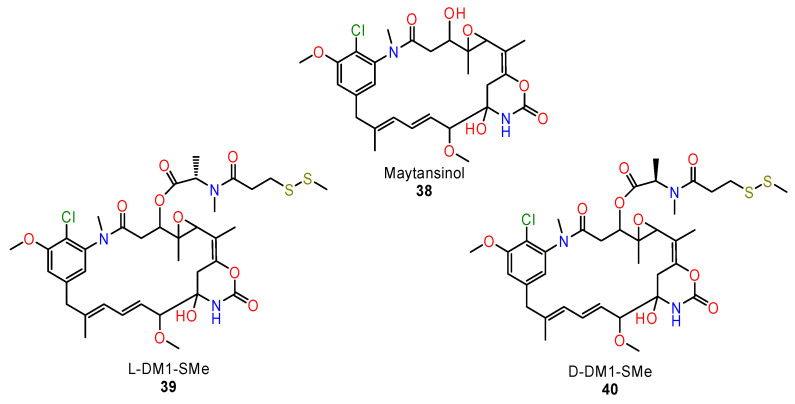
The structures of chiral derivatives of maytansine.

Based on these results, Li et al. [112] determined the high-resolution crystal structure of the tubulin complex with maytansinol and two stereoisomers of C3-ester side-chain derivatives D-DM1-SMe and L-DM1SMe. (**38**–**40**) (Figure 17).

**Figure 18:**
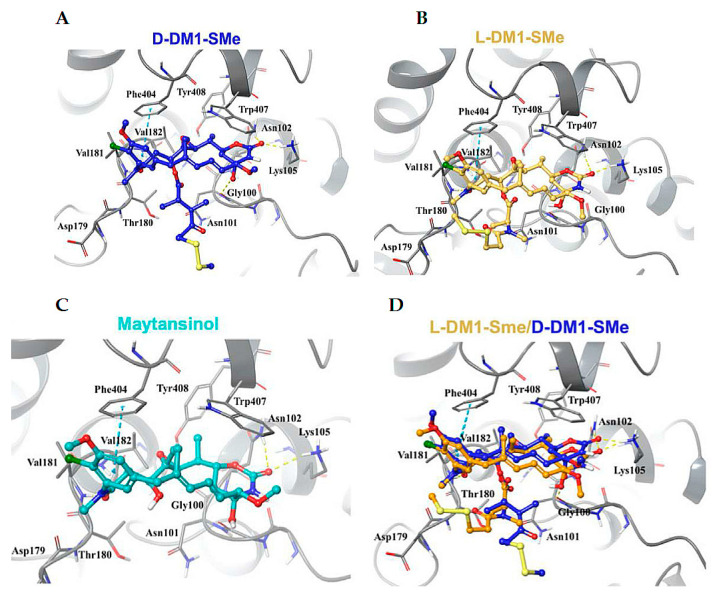
(**A**) Interactions between tubulin (grey ribbons and grey sticks) and D-DM1-SMe (yellow sticks); (**B**) interactions between tubulin (grey ribbons and grey sticks) and L-DM1-SMe (blue sticks); (**C**) interactions between tubulin (grey ribbons and grey sticks) and maytansinol (cyan sticks); (**D**) overlay of the binding poses of L-DM1-SMe (yellow sticks) and D-DM1-SMe (blue sticks). Reprinted with permission from Ref. [112]. Copyright© (2021), Elsevier.

The study of crystal structures revealed differences at the C3 side chain in D-DM1-SMe and L-DM1-SMe. The carbonyl oxygen atom of the ester moiety and the tail thiomethyl group at the C3 chain of L-DM1-SMe create strong intramolecular interactions with the hydroxyl at position 9 and the benzene ring, respectively, fixing the bioactive conformation and enhancing the binding activity. The C3 side chain of D-DM1-SMe is swung to the opposite direction, thereby losing the ability to create intramolecular interactions. The conformational differences may provide an explanation for how the chirality of the methyl group at the C3 position affects the anticancer activity (Figure 18).

## 6. Proteasome Inhibitors

The ubiquitin–proteasome pathway is the most important intracellular protein degradation system, and it is involved in processes such as apoptosis, cell survival, cell-cycle progression, DNA repair, and antigen presentation, among others. The proteosomal system includes different kinds of enzymes, which are modified by binding of several regulatory complexes to the core particle (the 20S proteasome) [113].

Inhibitors of the 20S proteasome (targeting the 20S catalytic particle) are an important class of drugs for the treatment of liquid tumours, such as multiple myeloma and mantle cell lymphoma, and they are being investigated for other diseases as well [114]. Bortezomib was the first proteasomal inhibitor to be approved by the US Food and Drug Administration. Carfilzomib and Ixazomib have recently been approved, and more drugs are in development [115]. However, these protease inhibitors have not demonstrated sufficient activity against solid tumours, and peripheral neuropathy is a dose-limiting toxic side effect for their clinical use [116].

Non-peptide inhibitors targeting different components of the proteasome system appear to be a promising alternative for the treatment of solid tumours. Anchoori et al. [117] presented the development of novel derivatives targeting the 19S regulatory particle unit which contains the ubiquitin receptor RPN13, RA 183, and RA375.

**Figure 19:**
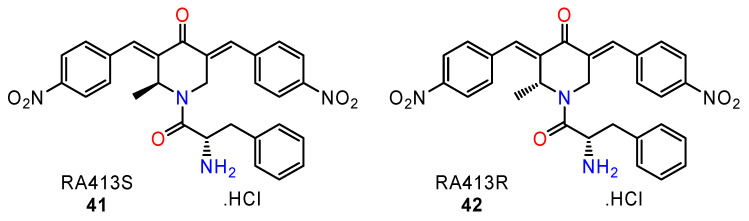
The structure of chiral proteasome inhibitors (**41**–**42**).

The preparation of new derivatives was rationalised. To improve their specificity and potency, several libraries of molecules were generated to probe the pharmacophore of the benzylidenepiperidone core unit and to identify the active compounds. Based on these findings and molecular modelling data, they introduced a methyl group at the ring carbon atom next to the nitrogen, and thus prepared chiral derivatives of perspective RPN13 inhibitors (**41**–**42**) (Figure 19).

The docking studies suggested the potential for differing RPN13 binding and cytotoxicity potencies of the racemic form R414 and the (*S*)-isomer R413S, and for weaker toxicity for R413R. Consequently, each form of active compound was synthesised and tested against several ovarian cancer cell lines. The cytotoxicity studies confirmed the theoretical predictions. RA413S was 5-fold more cytotoxic for HeLa cells than RA413R (23 nM vs. 172 nM). Similar phenomena appeared also in additional cell lines derived from ovarian cancer (e.g., SKOV3, TOV21G) and cervical cancer (HeLa, CaSki, SiHa). The cytotoxicity of RA413S for normal human cells was much weaker (IC_50_ > 100 nM).

The mechanism of antitumour activity for the active (*S*)-isomer RA413S and the racemate was further evaluated. The cancer cell toxicity was associated with improved binding to RPN13 lysates, ATP depletion, mitochondrial damage, oxidative stress, and glutathione and NF-κB inhibition [117].

## 7. PPARs Proliferator

Peroxisome proliferator-activated receptors (PPARs) belong to the group of nuclear receptors. They exist in three different isoforms: PPARα, PPARβ, and PPARγ, and are mainly produced in brown adipose tissue, gut, immune cells, liver, kidney, heart, and other tissues. PPARs play major regulatory roles in energy homeostasis and metabolic function by activation of fatty acid metabolism and stimulation of glucogenesis [118]. The modulatory function of PPARs-α and -γ is evident in immunity inflammation, vascular functions, cellular proliferation, differentiation, development, and apoptosis [119].

PPARs have become interesting therapeutic targets for the treatment of various diseases—dyslipidaemia, type 2 diabetes, cardiovascular diseases, obesity, cancer, and metabolic diseases [120]. PPAR modulators, including agonists and antagonists, could represent a novel strategy for preventing and treating multiple types of cancer [121]. The antitumour effect of PPARα and PPARγ in various types of cancers, both in laboratory and in clinical settings, have been recently reviewed [122,123]. The synthesised PPARγ modulators thiazolidinediones (TZDs), also known as glitazones, are involved in clinical phase trials for the treatment of prostate cancer, liver cancer, melanoma, and lung cancer.

In order to diminish the side effects of TZD treatment, novel PPAR ligands with different molecular scaffolds are being developed [124].

**Figure 20:**
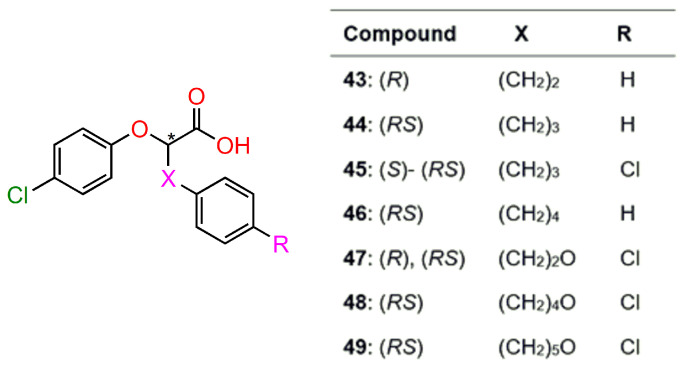
The structure of selected phenoxyacetic acid derivatives prepared as racemate and single enantiomers (**43**–**49**). * stereogenic centre.

Sabatino et al. [125] prepared and biologically evaluated new chiral derivatives of phenoxyacetic acid, acting as PPARγ partial agonists (**43**–**49**) (Figure 20). Their antiproliferative activity was evaluated in colorectal carcinoma cell lines (HT-29 and CRC). All compounds exhibited an antiproliferative effect in the range of 31–82% of residual vitality; with respect to the 60 % produced by the full i PPARγ agonist rosiglitazone. The compounds **45** (*RS*)-, (*S*)-isomers, and **49** (*RS*) forms were subjected to further evaluation since they combine the best antiproliferative activity (31–47% of residual vitality) and a limited *trans* activation (efficacy ranging between 55% and 65%) in comparison with the effects of all other compounds.

**Figure 21:**
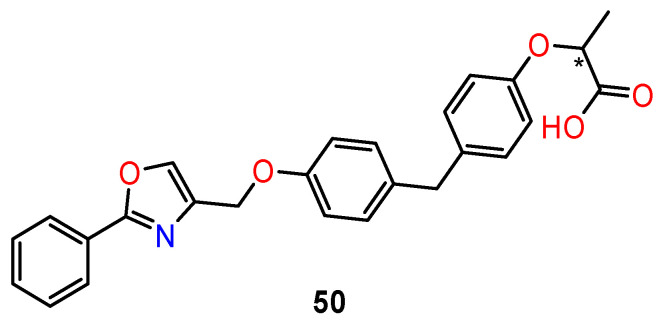
The basic structure of chiral diphenylmethane derivatives as PPARα/γ agonists. * stereogenic centre.

Piemontese et al. [126] designed a new class of dual PPARα/γ agonists based on the 2-oxy-propanoid acid moiety linked to diphenylmethane (**50**) (Figure 21). This structural skeleton is an active pharmacophore for the activation of PPARα/γ subtypes. Prepared diphenylmethane derivatives were tested for their agonist activity towards the human PPARα, PPARβ, and PPARγ subtypes. The highest activity was obtained in R1 compounds (47% activation compared to reference compounds). Single enantiomers were prepared to evaluate the influence of configuration on receptor activation. Unexpectedly, both enantiomers of R1 displayed similar activity towards all PPAR subtypes. To rationalise this effect, docking experiments were performed. The docking experiment predicted that both (*S*)-1 and (*R*)-enantiomers favourably bind to the PPARγ ligand-binding domain, adopting a similar U-shaped configuration that wraps around H3. In case of PPARα, both enantiomers fit the PPARα pocket well. The carboxylate head groups form the well-recognised H-bonding network with residues Y464, Y314, and S280, which is supposed to be critical for PPARα ligands’ activity.

The antiproliferative activities of racemate and both enantiomers were evaluated against HT-29 cells, with the (*S*)-enantiomer eliciting a more robust activity than the (*R*)-enantiomer. The alternative antiproliferative pathways were tested. The ability of the compounds to inhibit cell proliferation in colon cancer lines seems to be due to downregulation of Wnt/β-catenin signalling which is overexpressed in the majority of colorectal cancers. The (*S*)- and (*R*)-enantiomers strongly influenced mitochondrial function, as they activated the carnitine shuttle system through upregulation of the carnitine/acylcarnitine carrier and carnitine palmitoyl-transferase genes [126].

## 8. Conclusions

Chirality can be considered one of the major topics in the design, discovery, development, and marketing of new drugs. Chirality plays an important role for biological activities, so when a chiral centre is present in a drug, both enantiomers must be studied for the evaluation of their pharmacological properties. One enantiomer of a chiral drug may be a medicine for a particular disease, whereas another enantiomer of the same molecule may not only be inactive but can even be toxic.

This review outlines a variety of some recent examples of structurally diverse natural anticancer chiral compounds and their analogues exhibiting different mechanisms in their anticancer effect. The present survey represents up-to-date studies of the difference in biological activities between single enantiomers of anticancer agents and their racemic mixtures. The influence of stereoselectivity on anticancer activity is difficult to generalise, as it is manifested specifically for each individual chiral compound as well as in dependence on the type of cellular targets. The stereospecificity of new anticancer agents manifests itself in the cytotoxicity effect at the cellular level or in the interaction with subcellular structures. The awareness of the stereochemistry of anticancer compounds can help to understand some critical processes underlying their toxicity towards cancer cells and can provide a rational basis for the design of new antitumour drugs.

## Data Availability

Not applicable.
